# Supplementary material for: Are carbon emissions trading and green financial instruments synergistic? -Comprehensive quantitative research based on content analysis
Source: PLoS One. 2024 Mar 7;19(3):e0298601. doi: 10.1371/journal.pone.0298601 (PMC10919601; doi:10.1371/journal.pone.0298601)
Supplement: S1 Data — (ZIP) [file pone.0298601.s001.zip › Data/地级市Z_F工作报告.zip!/─┌├╔╣┼/╬┌║ú╩╨/└┤╫╘╣1⁄2╓┌║┼í╛┬φ┐╦╩2╛▌═°í┐_2011.docx]

各位代表：

　　现在，我代表市人民政府向大会作工作报告，请连同《乌海市国民经济和社会发展第十二个五年规划纲要（草案）》一并审议，并请各位政协委员和其他列席会议的同志提出意见。

　　一、2010年工作总结

　　2010年，是实施“十一五”规划的最后一年，也是我市推动科学发展取得显著成效的一年。一年来，在自治区党委、政府和市委的正确领导下，我们深入贯彻落实科学发展观，团结带领全市各族人民，抢抓机遇，奋力拼搏，真抓实干，圆满完成了市七届人大三次会议确定的各项目标任务，主要经济指标增速均居自治区前列。预计地区生产总值完成389亿元，按可比价计算增长21%。地方财政总收入完成68亿元，增长26%。固定资产投资完成238亿元，增长31%。城镇居民人均可支配收入19700元，增长12%；农区居民人均纯收入9200元，增长12%。社会消费品零售总额完成71亿元，增长18.5%。

　　经济结构调整力度加大，产业转型升级迈出新步伐。工业经济总量快速扩张，预计177户规模以上工业企业完成增加值243亿元，增长25%，增速居全区第3位，产值过10亿元工业企业达到10家，税收过千万元工业企业36家。能源、化工、建材、冶金等优势产业进一步壮大，原煤产量达到2900万吨、洗煤2900万吨、焦炭630万吨、PVC75万吨、水泥190万吨。煤焦化、氯碱化工两大基地形成规模优势，产业产品链条进一步延伸，产业规模、技术装备、节能、环保等方面已处于国内领先水平。在建和已建成百万吨焦化项目18个，其中建成投产11个，西部煤化、万晨能源等13个百万吨重介洗煤和神华能源甲醇、黑猫煤焦油深加工、华清焦炉煤气综合利用等项目顺利投产。中联化工PVC项目前期工作基本完成，盛远塑胶PVC软制品项目开工建设，海亮PVC管材型材项目建成投产。君正、赛马水泥和包钢万腾钢铁节能技改项目建设进展顺利，神华能源发电项目和京海电厂投入运行。装备制造、生物制药、新能源等非煤、非本地资源接续替代产业实现历史性突破，内蒙古华油、陕汽重卡、东北制药、上海利康、恒业成有机硅、腾龙精细化工等一批重大产业转型项目开工建设。强化园区建设，投入6.7亿元，进一步完善经济开发区水、电、路等配套基础设施，乌达经济开发区污水处理厂、海南区污水处理厂投入运行，园区集聚力和承载力明显提升。继续开展企业素质提升工程，企业整体形象和综合竞争力不断增强。进一步加大上级资金争取力度，到位各类资金25.5亿元。招商引资成效显著，实施招商引资项目112个，到位资金170亿元，增长39.3%；外贸进出口总额达到600万美元。

　　城建投入力度空前，城市面貌呈现新变化。以承办自治区“十二运”为契机，全面提升城市规划建设管理水平。启动编制乌海及周边地区城镇总体规划、三区控制性详细规划和环乌海湖概念性规划。黄河海勃湾水利枢纽工程全面开工建设，导流明渠工程竣工，具备截流条件。全年基础设施建设投入74亿元，增长58%。滨河新区一期城市功能进一步完善，滨河西区规划建设快速推进，乌海书画院、神华研发中心综合楼、五星级酒店等一批标志性建筑进展顺利。城市服务功能进一步完善，全面完成居民天然气置换工作，新建改造热力站17座，铺设供热主管网15.4公里，新增供热面积200万平方米。改革城市水资源管理运营体制，组建市水务集团公司。新建改造给排水管网39公里，供水水质合格率达97%以上。大力实施景观亮化工程，完成203处单体建筑及景观带亮化，新建改造路灯4000基。城市快速交通网络基本形成，新建改造海拉路、海北大街、海达街、乌兰淖尔环湖道路、公乌素街等34条街路，京藏高速乌海互通立交桥、林荫大道公铁立交桥、三区城际快速通道、110国道连接线和机场路改造等工程建成通车，海公铁路扩能改造全线贯通。完成机场航站楼和飞行区扩建，实现了北京直飞，增开上海航线，航线增至5条，空港旅客吞吐量超18万人次。城市更新改造力度加大，全年完成拆迁建筑面积81.3万平方米，收储土地600万平方米，新建续建410万平方米，城镇人均住房面积达到31平方米，三区面貌明显改观。生态园林建设力度加大，建成自治区级园林城市。青山翰墨园、神华墨玉广场、法制广场、运动公园等8个主题公园广场建成开放，乌达区人民公园完成改造，海南区人民公园启动建设。实施带状公园、街心游园、滨河景观带等绿化工程50多项，城区绿化覆盖率达到34.46%，人均公园绿地面积11.86平方米，高于全国平均水平。完成甘德尔河整治一期工程，实现河道蓄水，音乐景观喷泉投入使用。大规模开展城市环境综合整治，城市整体形象不断提升。

　　生产性服务业加快发展，第三产业规模层次实现新突破。预计第三产业增加值完成115亿元，增长13.5%，第三产业固定资产投资完成96亿元，增长60%，吸纳就业14.1万人，占就业比重的53.8%。围绕建设自治区西部物流中心，启动建设呼铁众利、如意俊安、海勃湾物流园等一批物流项目，呼铁同洲物流园一期和铁神物流园二期建成运营。通用时代广场、维多利财富广场等项目建设进展顺利，第一粮库退城进郊建设项目基本完成。房地产开发完成投资25亿元，商品房施工面积382万平方米，销售面积102万平方米。金融业实现新突破，乌海银行进一步做大做强，内蒙古银行、鄂尔多斯农村商业银行在我市设立分支机构，黄河村镇银行投入运营，新增小额贷款公司9家，全市贷款余额353亿元，新增贷款110亿元。旅游业发展步伐加快，甘德尔山生态文明景区、乌海博物馆建设进展顺利，旅游业总收入达到6.4亿元，增长17.6%。

　　 配套政策趋于完善，城乡一体化步入新阶段。制定实施了城乡一体化居民就业安置、住房、社保、低保、对口帮扶、耕地流转6个配套办法和库区征地、移民安置相关政策，农区居民生活保障水平不断提高，在就业、低保、社保、住房等方面享受比城市居民更加优惠的待遇。葡萄、蔬菜、花卉苗木优势产业进一步发展壮大，汉森葡萄酒庄园、吉奥尼葡萄庄园、农业科技示范园、云飞农科公司等16个投资千万元以上在建龙头项目进展顺利，葡萄种植公司化比例达到94%以上，5家农牧业产业化龙头企业通过有机认证。销售收入百万元以上加工企业实现销售收入2.8亿元，增长20%。全年争取上级各类支农资金2000万元，实施了一批整村推进、扶贫开发项目，开工建设农区居民安置楼48栋。进一步落实支农惠农政策，发放各类补贴资金1300万元。预计第一产业增加值完成3.5亿元，增长12.5%。

　　节能减排强力推进，生态环境建设取得新进展。继续加大落后产能淘汰力度，淘汰水泥65万吨、电石16万吨、铁合金9万吨、焦炭36万吨、玻璃34万重量箱。严格落实节能减排工作责任制，推动现有企业节能技术改造和清洁生产，推广使用煤矸石、粉煤灰砖等新型墙体建筑材料，完成建筑节能改造20万平方米，全年单位GDP能耗降低6.31%，二氧化硫和化学需氧量分别削减到8.6万吨和0.8万吨，圆满完成“十一五”节能减排目标任务。加大矿产资源开发综合整治力度，露天采矿区二次扬尘污染得到有效缓解。生态建设力度加大，投入6亿元，实施机场路、110国道、包兰铁路乌海段、乌达西山、海南环城绿化带等26项林业重点工程，完成造林面积10万亩，森林覆盖率达到17.49%。深入推进新一轮“蓝天工程”，海勃湾城区优良天数达到295天，乌达、海南城区分别达到236天和230天。

　　富民惠民力度加大，民生工作再上新台阶。财政支出58%用于民生事业，承诺为民所办十件实事全部完成。保障性安居工程稳步推进，投入26.8亿元，开工建设各类保障性住房33139套、240万平方米，搬迁入住6894户，低保无房户全部实现实物配租，发放租金补贴357万元，15平方米以下低保户住房实现应保尽保。创业带动就业成效明显，建成创业孵化基地6个，全年新增就业8856人，下岗失业人员再就业5500人，发放小额担保贷款6336万元， 1773人成功创业，创业活动对就业的贡献率达到15%，城镇登记失业率控制在4.3%以内，动态消除了“零就业”家庭。社会保障力度加大，原国有、集体、劳服、农垦企业职工全部纳入养老保险统筹，市区两级又安排1亿元资金，资助“零社保”低保家庭、库区移民、残疾人、精神病人、优抚对象等困难群体1.88万人参加养老保险。争取神华集团、自治区政府资金1.18亿元，为神华乌海能源公司、市属国有企业解除劳动关系人员补贴续保缴纳养老保险金，2.9万人受益。全市离退休人员取暖费补贴标准和企业退休人员平均养老金标准分别提高到650元和1364元。全市各类关闭破产企业退休人员、困难企业职工全部纳入职工医疗保险，职工和居民医疗保险报销限额分别提高到28万元和9万元，职工医保银联卡正式启用。增加外地知名医院为医疗定点医院，群众就医选择权得到有效保障。工伤、失业、生育保险覆盖面不断扩大，参保人数分别达到8.53万人、10.5万人和12万人。社会救助体系进一步完善，居民最低生活保障标准提高110元，达到360元，月人均补差提高34元。为企业退休人员病亡遗属每人每月增加200元生活困难补贴。发放贫困大学生救助金和市长助学金112万元，400多名贫困学生顺利入学。为特困群众发放医疗救助金800万元，1万多人受益；发放一次性价格临时补贴资金720万元，2.4万人受益。农区居民人均纯收入、退役士兵自谋职业补助、五保供养和孤儿供养标准居全区第1位，低保标准、医保报销比例居全区第2位，其他民生指标均居自治区前列。社会福利事业进一步发展，市儿童福利院、流浪儿童救助保护中心开工建设。投入7476万元，完成社区用房新改扩建项目16个。免费为适龄聋儿实施人工耳蜗植入手术。积极推进“白内障无障碍市”创建工作，为500名患者免费实施复明手术。制定出台少数民族优惠政策，惠及少数民族群众3.15万人。计划生育家庭奖励扶助政策扩大到全市范围，奖励扶助金提高到2000元。继续开展“一杯奶”生育关怀行动，启动实施“公共卫生服务九项”、“优生八项”和“两癌”普查等惠民工程。加快公交事业发展，市政府投入3000万元购置新能源公交车100辆，新开延伸公交线路5条，增加公交班次，降低票价，海南长途汽车站投入运营，群众出行更加便捷。

　　社会事业进一步发展，和谐稳定开创新局面。加强教育基础设施建设，全市累计投入建设资金3.6亿元，全面推进中小学校舍安全工程，累计开工校舍60栋、建筑面积28万平方米。滨河中学和市特殊教育学校新校区投入使用，建成市职业技术学校实验实训楼和市十中综合楼。高中教学质量稳步提高，全市高考二本以上上线率达到36%，比自治区平均录取率高出10.5个百分点。高度重视幼儿教育，制定出台加快幼儿教育发展政策意见，投入2060万元，新建3所标准化幼儿园。新购置一批教育教学设备，职业教育、民族教育和特殊教育办学条件进一步改善。加大校园安全人防、物防、技防投入力度，学生人身安全得到有效保障。医药卫生体制改革稳步推进，实行基层公立医疗机构药品零差率销售。新建社区卫生服务中心8个，市人民医院内科楼、市妇儿医疗预防保健中心建设进展顺利，市蒙中医院正式运营。投入近6000万元购置一批先进医疗设备，与多家国内知名医院建立良好合作关系，成功开展一批高技术医疗项目，医疗和公共卫生服务水平大幅提升。科技创新能力稳步提高，与中科院北京分院、天津大学、北京化工大学、内蒙古工业大学达成战略合作协议，产学研基地成果转化应用步伐加快，高新技术产业增加值率、万名就业人员发明专利拥有量两项指标位居全区前列。人才工作不断加强，全市企事业单位引进各类人才2755名。文化、体育事业蓬勃发展。新建煤炭博物馆、葡萄酒博物馆、青少年宫和5个文化站、31个草原书屋。大力推进书法城建设，成功举办首届国际书法产业博览会，近30个国家的大使、参赞、著名书法家和专家学者400余人参会，500余件艺术精品参展，近2000名展销商积极参与，书画、奇石、会展等文化产业不断发展壮大。精神文明创建活动扎实开展，组织“万人看乌海”活动，近万名群众感受城市新变化。全面启动“十城联创”，成功举办全市第二届两个文明建设经验交流会，全社会文明程度进一步提高。高水平、高质量承办自治区第十二届运动会，成功组织400多场赛事活动，600多名志愿者积极投身服务，接待运动员、裁判员和来宾万余人，赛事服务和后勤保障等各项工作实现零失误、零差错目标。以自治区“十二运”为契机，大力推进体育事业发展，市体育中心、网乒中心等一批大型体育场馆投入使用，公共体育设施建设达到自治区先进水平。自治区“十二运”和国际书法产业博览会的成功举办，充分展示了我市经济社会发展成就和城市建设巨大变化，充分展示了我市人民奋发进取、和衷共济、开放包容的城市精神，极大提升了我市的知名度和美誉度。

　　民主法制建设稳步推进。自觉接受人大法律监督、工作监督和政协民主监督，主动向市人大、政协报告、通报工作。办理人大代表议案、建议200件和政协委员提案163件。认真听取各民主党派、工商联、无党派人士意见，支持工、青、妇等人民团体开展工作。加强基层民主建设，全面完成“五五”普法验收工作，全社会民主法制意识进一步增强。稳步推进新一轮政府机构改革和行政管理体制改革，强化政务服务中心建设，清理行政审批事项175项，取消行政事业性收费70项，政府效能稳步提升。推进公共资源交易中心改革，工程招投标、产权交易和政府采购等工作进一步规范。加强对政府投资项目、预算执行情况和民生领域资金审计工作，保障了资金安全有效运行。严格执行“一岗双责”等各项安全生产责任制，加强安全生产基层基础建设，强化技术支撑，健全隐患排查治理、危险源监控制度，加大煤矿、危化企业等重点领域安全生产监察执法力度，安全生产责任制得到有效落实。加强食品药品监管长效机制建设，食品药品安全保障水平进一步提升。全力推进社会矛盾化解、社会管理创新、公正廉洁执法三项重点工作，解决了一批历史遗留问题和关系人民群众切身利益的突出问题，严厉打击各种违法犯罪，社会总体保持和谐稳定。编制完成“十二五”规划。双拥工作成果继续巩固，荣获自治区双拥模范城“六连冠”。第六次全国人口普查完成入户登记工作。广播电视网络实现全覆盖。国防建设、民兵预备役、档案史志、侨务外事、人防、气象、防震减灾、新闻出版、无线电管理、红十字和老龄事业等工作均取得新成绩。

　　 二、“十一五”工作回顾

“十一五”时期，是我市建市以来经济增长最快、城市变化最大、人民群众得到实惠最多的五年。五年来，面对国际金融危机严重影响和节能减排的强大压力，我们上下齐心、攻坚克难、积极进取，全市经济社会继续保持健康快速发展，取得显著成绩。

——经济总量实现倍增，超额完成再造“两个乌海”目标。地区生产总值是“十五”期末的3.24倍，年均增长26.5%，人均GDP突破1万美元。工业、农业和第三产业增加值分别是“十五”期末的3.9倍、1.8倍和2.6倍。地方财政收入是“十五”期末的3.75倍，年均增长30%。固定资产投资是“十五”期间的2.6倍，超过“十一五”前历年投资总和。这些成就的取得，标志着我市经济社会发展进入了一个新的历史阶段。

　　——两大基地建设成效明显，经济转型实现重大突破。工业经济在调整结构中扩张总量，在做大总量中加快转型升级。坚持“关小上大、扶优汰劣”，大力推进产业结构调整，累计关停小企业1500多户，100万吨以下焦化企业全部关停，万元GDP能耗从“十五”期末的6.75吨标煤下降到4.84吨标煤，二氧化硫排放量在GDP翻一番的情况下减少3.46万吨。加快产业多元、延伸、升级步伐，煤焦化、氯碱化工两大基地规模优势进一步显现，一批投资大、带动力强的大企业大集团迅速发展壮大，非煤、非本地资源等接续替代产业取得突破性进展。规模以上工业企业由147户增加到177户，户均产值由不到1亿元提高到3亿多元，规模以上工业增加值占地区生产总值的比重达到63%，对全市经济增长贡献率提高到73%。第三产业取得长足发展，生产性服务业发展迅速，一批大型物流项目开工或运营。金融业发展步伐加快，多家银行在我市设立分支机构。旅游业取得历史性突破，甘德尔山生态文明景区等一批旅游景区开工建设。坚持“转移、减少、集中、富裕”的发展思路，五年转移农区居民2.4万人，流转土地1.4万亩，全市54个行政村整合为13个行政村和6个涉农社区。不断完善政策保障措施，一产与二、三产业融合互促步伐加快，城乡一体化发展向纵深推进。

　　——城市面貌发生巨大变化，区域中心城市集聚辐射功能进一步增强。高标准、高起点修编完成新一轮城市总体规划、土地利用总体规划和重点区域规划，实施了城市功能设计、特色街路、园林景观等专项规划编制工作。围绕“三进一靠”，大力推进滨河新区建设和旧城区扩容提质，城市框架全面拉开，城区面积由2005年的37.5平方公里增加到53平方公里。依托库区规划建设的滨河一期基本建成，一批基础设施建设项目相继完工。全市供气、供热普及率和生活垃圾、城市污水处理率分别达到69.5%、80.8%、81%和89.8%。一批重点交通项目建成通车，区域立体交通网络初步形成。绿化、美化、亮化、净化工程深入推进，区域中心城市承载力和辐射力进一步增强。

　　——发展成果普惠民生，居民收入实现翻番。坚持“以人为本、富民优先”，财政投入更多向民生倾斜，城乡居民收入实现翻番，农区居民人均纯收入连续6年位居全区第一。实施惠及5.1万户、15.1万人的棚户区居民搬迁改造工程，采煤沉陷区搬迁改造工程全部完成。大力推进就业、社保、医保、低保等民生事业，惠及全民的民生保障体系初步形成，各项民生指标均位居自治区前列。不断加大科技、教育、文化、卫生、体育、安全等社会事业投入，和谐社会建设取得明显成效。

　　各位代表，过去五年我们所取得的成绩和进步，是自治区党委、政府和市委正确领导的结果，是全市各族人民共同团结奋斗的结果，在此，我代表市人民政府，向在各个领域和岗位上辛勤工作的各族干部群众，向给予我们大力支持的人大代表、政协委员、各民主党派、工商联和社会各界人士，向驻市部队、武警消防官兵、公安干警和各方面朋友，表示崇高的敬意和衷心的感谢！

　　在看到成绩的同时，我们也清醒地认识到，我市经济社会发展还存在诸多困难和问题。主要是：经济总量偏小，产业发展对资源的依赖偏重，接续替代产业发展不足，服务业相对滞后，结构调整和经济转型任务繁重；城市基础设施还不完善，管理水平不高，区域中心城市辐射带动能力有待进一步提升；资源、环境约束趋紧，环境保护和生态建设仍需不断加强；民生社会事业与经济发展不够协调，物价监管、养老服务、物业管理、“菜篮子”工程建设等还不能完全满足群众需求，社会管理和公共服务能力需进一步提高；政府职能转变和作风建设与建设法制型、服务型政府的要求还有差距，行政效能有待进一步提高。对此，我们将予以高度重视，在“十二五”时期，采取切实有效措施着力加以解决。

三、“十二五”工作思路与目标

　　“十二五”时期是我市实现经济和城市转型、建设自治区西部区域中心城市的关键时期。根据市委五届十二次全委会议精神，“十二五”时期政府工作的总体思路是：以邓小平理论和 “三个代表”重要思想为指导，深入贯彻落实科学发展观，以加快转变发展方式为主线，以结构调整为主攻方向，以改革开放和科技创新为动力，以保障和改善民生为根本，加快推进经济转型、城市转型，努力把我市建设成为“人口产业集聚、生态环境宜居、社会民生普惠和谐”的自治区西部区域中心城市。

　　主要预期目标是：地区生产总值年均增长21%，突破1000亿元；地方财政总收入年均增长22%，达到180亿元，力争达到190亿元；全社会固定资产投资确保五年累计完成1500亿元，力争完成1700亿元；居民人均可支配收入年均增长14.8%，达到4万元以上；节能减排完成自治区控制性指标；城镇登记失业率控制在4.2%以内；人口自然增长率控制在6‰以内。

　　围绕上述总体思路和发展目标，《乌海市国民经济和社会发展第十二个五年规划纲要（草案）》提出了“十二五”时期我市经济社会发展的主要任务和工作措施，重点要扎实做好以下六个方面的工作：一是加快经济转型，构筑现代产业体系；二是推动城市转型，增强辐射带动能力；三是实施创业就业、收入倍增、社会保障、住房安居、人口素质、人才强市、法治文明、公共安全八大民生工程，建设和谐乌海；四是大力发展社会事业，提升基本公共服务水平；五是加强生态环境保护，增强发展承载力；六是深化改革扩大开放，激发发展活力。这些重点工作的具体内容已全部体现在了《纲要（草案）》中，本次会议通过后，市政府将以积极的态度和务实的作风，认真组织实施。

四、2011年的主要工作

　　2011年是实施“十二五”规划的开局之年，也是加快推进我市经济和城市转型、打造自治区西部区域中心城市的关键一年。做好今年的工作，保持经济社会发展良好势头，对于开创我市“十二五”时期经济社会发展新局面意义重大。

　　主要预期目标是：地区生产总值增长22%，完成475亿元；地方财政总收入增长22%， 完成83亿元；固定资产投资增长20%，完成285亿元；城镇居民人均可支配收入增长13%，达到22260元；农区居民人均纯收入增长15%，达到10580 元；社会消费品零售总额84亿元，增长18%；节能减排、居民消费价格指数和城镇登记失业率控制在自治区下达指标以内；人口自然增长率控制在6‰以内。

　　实现上述目标，要重点抓好八个方面的工作：

　　（一）以扩总量、调结构为中心任务，加快经济转型步伐

　　加大固定资产投资力度，进一步优化投资结构，做大做强优势特色产业，以增量促调整，以调整促转变，推动经济又好又快发展。

　　一是加快打造“一城两基地”。推动自治区西部精细化工城建设，着力促进煤焦化、氯碱化工产业融合互促，产业链相互衔接。重点打造以资源精深加工、产业上下游协作配套和循环发展为纽带的精细化工产业链，构建精、细、深的加工体系。加快推进东源科技1，4-丁二醇、恒业成有机硅、腾龙精细化工、东北制药、西部锆业、明海锆业等项目建设。着力提升“两大基地”建设水平，全力打造全国重要的煤焦化生产、加工基地和交易中心及全国最大的PVC生产基地和交易中心。开工建设佳鑫、华信等重介洗煤项目，建成宏阳、家景等7个百万吨级焦化项目，加快华油焦炉煤气制天然气、神华22万吨硝铵等深加工项目建设。积极抓好中联化工50万吨PVC、40万吨烧碱、80万吨电石、100万吨白灰和盛远8万吨PVC软制品等项目建设。大力推进产业多元，积极发展非煤、非资源型产业，突出抓好包钢万腾钢铁节能技改、陕汽5万辆天然气重卡汽车、庞大专用汽车改造、蒙达一期30万条轮胎、斯凯夫高档油漆、隆兴佰鸿科技LED节能照明二期等产业转型项目建设。

　　二是大力推进科技创新，深入推动“两化融合”。强化企业技术创新主体地位，进一步加强与科研院所合作，充分发挥中试平台作用，积极支持宜化、君正等企业建设研发中心，促进科技成果转化，进一步提升产业发展水平。以我市被列为首批“国家级两化融合试验区”为契机，积极推进神华乌海能源、乌海化工、君正科技、内蒙宜化、黄河工贸5家企业信息化试点工作。

　　三是狠抓企业素质提升。扎实开展企业素质提升工程，引导企业以品牌、标准、服务和效益为重点，健全质量管理体系，提高企业科学管理水平和抵御市场风险能力，全面提升企业内在发展能力和外部竞争能力，力争用两到三年时间，使全市企业素质明显提升。积极扶持乌海化工、君正科技等企业尽快上市。

　　四是继续加大经济开发区基础设施建设力度，启动西来峰工业园区南区建设。新建千里山工业园区、君正集团、蓝星玻璃公司铁路专用线。围绕化工企业下游产品开发，重点配套完善供水、供电、供气、路网等相关设施。高起点设计西来峰工业园区南区产业体系，高水平规划园区功能布局，高标准建设园区基础设施，引领区域产业延伸和产业升级。

　　五是加大招商引资力度。全力对接在谈项目，确保尽快开工建设，切实提高项目履约率和资金到位率。主动承接先进地区产业转移，积极引进配套服务的深加工项目和非资源型产业转型项目，力争将我市建成西部地区承接产业转移示范园区。做好招商引资项目跟踪服务工作，争取年内引进到位资金185亿元。

　　六是大力扶持中小企业发展。密切关注和把握国家宏观经济政策，及时协调解决企业生产经营中遇到的困难和问题，确保经济平稳运行。认真落实自治区“促进中小企业政策落实年”各项措施，培育和引进中小企业，鼓励协作配套，引导集中布局，加快形成大中小企业协同共进的发展格局。积极做好统筹区域协调发展相关工作，在产业布局、产业协作、政策协同、生产要素配置等方面，主动加强与周边地区沟通联系，推动区域一体化发展。

　　（二）以乌海湖形成为契机，精心打造滨水宜居城市

　　着眼于打造滨水宜居城市，配合自治区编制完成《乌海及周边地区城镇总体规划》，高标准完成重点区域规划和控制性详细规划、环湖景观规划等专项规划。围绕环湖开发建设，加快启动乌海湖及周边绿地、水系、景观等基础设施建设，推动三个城区向乌海湖靠拢，实现适度集中、协调发展。进一步完善滨河一期功能，启动中心商业区建设，完成神华研发中心综合楼、五星级酒店等续建项目，新建市质量计量特种设备检验检测中心、市职工文化活动中心。加快推进黄河海勃湾水利枢纽工程建设，确保取得实质性进展。完成库区征地拆迁、规划工作，继续建设黄河护岸工程，全面启动滨河二期建设。进一步完善城市水系，改造完成甘德尔河景观二期工程，实施环海勃湾水系工程。加大三区城市棚户区拆迁改造力度，新建建筑面积130万平方米，启动海勃湾区海北新城建设。大力推进基础设施建设，建成供热站34座、配水厂4座，新打水源井33眼，铺设给排水管网96公里。加大城市交通基础设施建设，完成海勃湾区狮城街、乌达区滨河大道、海南区经七路等22条街路新建改造，开工建设110国道黄河大桥、跨乌海湖大桥、海南至乌达货运重载通道和海南至拉僧庙二级公路，建成海南区黄河路互通立交桥。

　　以更高标准、更大力度推进全国绿化模范城市和国家园林城市创建工作，完成市植物园和海南区人民公园改造，建成开放东外滩公园。大力开展街路、园区企业、住宅小区、机关单位等绿化美化工程，全年新增绿地面积580万平方米，人均公园绿地面积达到12平方米。重点抓好沿河、沿路、沿山、沿边等绿化景观建设，抓好库区护岸林、甘德尔山东坡绿化、乌阿边界乌兰布和沙漠锁边林带等28项绿化工作，完成造林绿化10万亩，森林覆盖率提高到21%。进一步加大城市管理力度，大力推进环境综合整治，对集贸市场、居民区、街路死角、拆迁改造区环境卫生进行集中整治，解决好商业区便道、街路两侧车辆乱停乱放问题，集中实施重点路段、区域、大型建筑亮化装饰工程。严厉打击损毁、偷盗公共服务设施行为。加强争做文明市民宣传教育，动员全社会自觉维护城市形象，努力营造整洁优美、秩序井然的城市环境。

　　（三）以生产性服务业为主导，促进第三产业提质增效

　　不断完善加快发展第三产业的政策措施。积极推进海勃湾物流园、呼铁众利、如意俊安、同洲物流园二期、锦达物流园建设，建成广东塑料交易所物流交收仓储中心。依托货运重载通道，突出抓好集汽车维修、货物配送、运输仓储于一体的公路物流业发展，积极推动二十五公里地区多功能物流园等一批物流项目建设。开工建设维多利财富广场和九龙国际广场，建成通用时代广场、万锦商厦。加快发展金融业，积极引进包头商业银行等金融机构在我市设立分支机构，力争成为自治区知识产权质押融资试点城市。突出抓好旅游业发展，制定出台旅游业发展规划和鼓励政策，加快推进甘德尔山生态文明景区建设，做好龙游湾湿地公园开发和乌海湖旅游项目前期工作，乌海博物馆年内投入使用。大力发展电子商务、连锁经营、特许经营等新型消费业态，提高服务市场化、社会化水平，增强区域辐射力和竞争力。继续推动“数字乌海”建设，组织实施应急管理、便民服务、社会治安和数字城管等一系列信息化应用项目。强化房地产市场监管，促进房地产业健康发展。积极发展家庭服务业，进一步规范物业管理行业，提高社区管理服务水平，方便居民群众生活。

　　（四）以落实政策措施为依托，加快推进城乡一体化发展

　　进一步落实城乡一体化发展各项配套政策，促进农区居民向城市有序流动，引导进城农区居民转变思想观念和生产生活方式，尽快融入城市生活，共创共享现代城市文明。通过信贷贴息、社保优惠、岗位补贴等扶持政策，鼓励企业优先吸纳农区劳动力就业，提高农区居民非农收入，年内转移农区居民9000人。加快农区土地流转，推进规模经营。加大对葡萄、蔬菜、花卉苗木产业扶持力度，大力发展休闲观光农业，支持市农业科技示范园、汉森葡萄酒庄、吉奥尼葡萄酒庄等一批龙头企业尽快做大做强。认真落实各项支农惠农政策，继续做好农机购置补贴工作。全力实施“菜篮子”工程，进一步加大资金投入力度，加强蔬菜基地和市场建设，扩大蔬菜种植面积，努力缓解居民吃菜贵问题，“十二五”期末蔬菜自给率达到50%。

　　（五）以加强节能减排、环境保护和安全生产为重点，着力改善地区发展环境

　　加大现有能耗企业节能管理力度，抓好工业、建筑、交通、公共机构等领域节能工作。加快污水、垃圾处理等基础设施建设，完成乌达、海南生活垃圾无害化处理厂和市污水处理厂二期工程。严把环保、用能准入关，继续保持项目环评、能评执行率和“三同时”率100%。深入开展矿产资源开发综合整治，重点抓好石灰石开采、煤矸石、劣质煤等固废污染和露天开采污染治理，有效缓解矿山企业二次污染问题。坚持节约集约发展，积极引进各种废弃物和再生资源利用技术，重点抓好粉煤灰、电石渣、煤矸石、焦炉煤气等固废和再生资源的回收利用，开工建设赛马二期日产4500吨水泥熟料配套低温余热发电等综合利用项目，提高资源综合利用水平，争取将我市列入资源型城市可持续发展试点城市。加强对土地利用状况评价与分析，进一步提高投资强度和土地利用强度。加强水源地保护和水质监测，保障居民饮水安全。深入推进新一轮“蓝天工程”和二氧化硫达标专项整治行动，中心城区空气质量优良天数超过295天，可吸入颗粒物浓度下降10％，二氧化硫年均值达到国家二级标准。

　　进一步强化安全生产责任制，完善安全生产目标考核，进一步抓好煤矿、非煤矿山、危化企业、建筑工地、消防、交通等行业和领域专项整治，加快推进煤矿安全质量标准化建设。建立完善应急救援体系，深入开展隐患排查治理，坚决遏制重特大事故，推动安全生产形势持续稳定好转。

　　（六）以解决就业社保住房为突破口，推动民生工作达到新水平

　　大力推进创业带动就业，加大政策引导和资金扶持力度，突出抓好高校毕业生、库区移民、残疾人、低保人群、计生困难户的就业创业服务工作。积极开展创建充分就业社区活动，城镇新增就业岗位9000个，开发公益性岗位1000个，动态消除“零就业”家庭。对下岗失业人员、进城农区居民等群体实行免费技能培训和创业培训，建成14个创业实训基地和孵化基地，年内培训1万人次。强化劳动保障监察，进一步完善有利于提高劳动者报酬的工资集体协商机制、决策机制、正常增长机制和支付保障机制，切实维护劳动者合法权益。依据国家政策继续提高企业退休人员基本养老金，月人均基本养老金提高10%左右。做好“五七工”纳入基本养老保险收尾工作，消除“零社保”家庭。进一步提高医疗保险待遇水平，启动居民基本医疗保险普通门诊统筹，城镇职工和居民基本医疗保险住院费用报销比例分别达到87%和70%以上。扩大生育保险覆盖面，将全市机关事业单位职工全部纳入生育保险统筹。继续提高最低生活保障标准和优抚对象保障标准。发放专项资金补贴卡，加大贫困居民在取暖、购房、入学等方面的救助力度。启动实施“金保工程”和市民健康信息化建设项目，实现社会保障基础数据网上运行和医疗基本信息“一卡通”运行。进一步提高独生子女父母奖励费，60岁以上老年人公交补贴增至100元。加快保障性安居工程建设，年内新开工保障性住房88.5万平方米，其中新建棚户区改造住房7529户、公共租赁住房1300户、廉租房1300户、限价房500户，有效解决中低收入家庭住房问题。大力发展社会福利和慈善事业，高度重视老龄事业发展，完善老年人服务体系，加快养老服务设施建设。启动“听力无障碍市”创建工作，建成市儿童福利院和流浪儿童救助保护中心。加快推进社区建设，新改扩建社区23个。大力发展公交事业，加快公交场站建设，增加公交站点和班次，开通海勃湾至蒙西和海南城区公交线路，逐步延伸乌达至乌斯太、海南至棋盘井公交线路，方便群众出行。新建乌海客运枢纽站，组建乌达、海南公交公司，实现市内公交一体化。采取各种有力措施，稳定物价。

　　（七）以加强书法城建设为抓手，全力打造城市特色文化品牌

　　进一步加强中小学书法教育和普及，完善书法家引进、培训和激励机制，积极推进书法进社区、进企业、进机关活动，建设老中青少梯次发展的书法人才队伍。加快推进乌海书画院建设。加强书法文化对外交流，继续开展“乌海书法万里行”活动，积极举办第二届国际书法产业博览会和全国产业职工书法艺术节等有影响力的书法会展，进一步扩大和提升乌海书法城品牌影响力。规划建设书法产品交易中心和书法文化创意产业园区，新建乌达区文化产业园区。大力促进书法理论探讨、创新及书法品评、鉴赏，建立多元化的文化产业投资机制，促进文房四宝、文博会展、书画奇石印章等产业发展。积极培育发展摄影、赛车、藏獒、信鸽、地方工艺品等产业。将书法文化元素融入城市建设，逐步用书法艺术规范和美化公共服务场所牌匾，城区主要出入地段设立鲜明的书法文化标志，在广场、主要街道、重点企业和重要窗口服务单位等场所设置具有书法特色的文化景观，三区各建一条书法文化特色街路。结合乌海湖和甘德尔山生态文明景区开发建设，推动书法文化、草原文化、黄河文化融合发展，不断提升城市内涵和品位，逐步将我市打造成为文化旅游城市。

　　（八）以促进社会和谐稳定为目标，全面提升社会建设水平

　　结合标准化学校建设，继续推进中小学校舍安全工程，新改扩建校舍3.74万平方米，建成市十中报告厅、教学楼和蒙古族中学生活服务楼，开工建设蒙古族中学教学楼、市十中体育馆和职业技术学院实训基地。加大对薄弱学校投入，促进义务教育均衡发展。高度重视学前教育，将学前教育经费列入市区财政预算，设立幼儿园发展专项资金1500万元，多渠道筹资新建10所标准化幼儿园。对公办普通高中教育、民族教育、中等职业教育学生实行全免费。推进职业教育发展，结合我市产业特点和用工需求，推动乌海学院和职业技术学校联合企业开展定向培养，不断提高职业教育办学规模和发展水平。继续深化教学改革，全面提高教育教学质量。完成国家教育考试标准化考点监控系统建设。加强校园安全教育管理，切实保护学生安全。抓好高层次、实用型人才引进和培养，满足本地发展需求。加快医药卫生体制和公立医院改革，建成市人民医院内科楼和市妇儿医疗预防保健中心，启动建设精神卫生中心综合楼，新购置一批先进医疗设备。扎实做好疾病预防控制工作，切实提高突发公共卫生事件的应急处置能力。加快培育广播电影电视文化发展，投入2000万元完成广电设备系统化、数字化、网络化升级改造。深入推进“十城联创”工作，广泛开展群众性精神文明创建活动，在海南区举办第三届全市精神文明建设经验交流会。设立专项资金，加强基层文化设施建设，支持群众文化艺术团体发展，丰富群众文化生活。积极承办区内外有影响力的体育赛事，举办全市中小学生运动会和全市运动会，提高体育场馆使用效率和社会效益。大力发展群众体育事业，广泛开展全民健身运动。

　　启动“六五”普法工作，推进依法治理和法治城市建设，依法严厉打击各类违法犯罪活动，积极推进“科技防控城市”和“平安乌海”建设，进一步增强人民群众的安全感。加强信访矛盾排查化解，畅通民意诉求渠道，妥善解决人民群众反映的热点难点问题，切实维护群众合法权益。全面加强应急管理，完善应急机制，及时妥善处置各类重大突发事件。进一步理顺食品药品监管体制，深入推进“食品放心城市”建设，切实保障全市人民饮食用药安全。进一步做好人口和计划生育、民族宗教、档案史志、双拥优抚、侨务外事、气象、防震减灾、无线电管理等工作。

　　切实加强政府自身建设。严格依法行政，建设法治政府。切实把依法行政作为政府工作的行为准则，严格依照法定权限和程序行使权力、履行职责。自觉接受人大及其常委会的法律监督和工作监督，执行人大及其常委会的决议、决定，主动接受政协的民主监督，办好人大代表议案、建议和政协委员提案，认真听取各民主党派、工商联、人民团体和社会各界意见。加强行政执法监督，落实行政执法责任制和过错追究制，促进严格执法、公正执法、文明执法。大力推行政务公开，认真贯彻《政府信息公开条例》，扩大公开范围，丰富公开形式。提高服务意识，建设效能政府。加快政府职能转变，调整优化机构设置，把政府的主要精力更多地集中到社会管理和公共服务上来。深化行政审批制度改革，集中清理行政审批事项。加大行政事业性收费清理力度，继续取消一批行政事业性收费项目，进一步优化发展环境。强化效能建设，严格行政问责，切实解决有令不行、有禁不止现象和慢作为、不作为、乱作为问题。加强电子政务建设，拓展网上审批服务，提升在线服务水平和效率。加强廉政勤政，建设廉洁政府。全面落实党风廉政建设责任制，严格执行反腐倡廉各项规定，加大审计监督和行政监察力度，严肃查处各类违纪违法案件，坚决纠正损害群众利益的突出问题和部门行业不正之风。教育和引导广大公务员紧紧围绕职能职责和工作目标，不断强化大局意识、责任意识和发展意识，始终保持奋发进取、干事创业的精神状态，不断开创各项工作新局面。

　　各位代表，2011年是“十二五”的开局之年，未来五年是我市推进经济和城市转型、建设自治区西部区域中心城市的关键时期，目标令人鼓舞，形势催人奋进，让我们在市委的坚强领导下，紧紧依靠全市各族人民，进一步解放思想，求真务实，真抓实干，为实现我们的宏伟目标而努力奋斗！
